# Acute Ischemic Stroke During the Convalescent Phase of Asymptomatic COVID-2019 Infection in Men

**DOI:** 10.1001/jamanetworkopen.2021.7498

**Published:** 2021-04-22

**Authors:** Tian Ming Tu, Christopher Ying Hao Seet, Jasmine Shimin Koh, Carol Huilian Tham, Hui Jin Chiew, Jasmyn Angon De Leon, Christopher Yuan Kit Chua, Andrew Che-Fai Hui, Shaun Shi Yan Tan, Shawn Sushilan Vasoo, Benjamin Yong-Qiang Tan, N. Thirugnanam Umapathi, Paul Anantharajah Tambyah, Leonard Leong Litt Yeo

**Affiliations:** 1 Department of Neurology, National Neuroscience Institute, Singapore; 2 Department of General Medicine, Khoo Teck Puat Hospital, Singapore; 3 Division of Neurology, Department of Medicine, National University Health System, Singapore; 4 Division of Neurology, Department of Medicine, Ng Teng Fong General Hospital, Singapore; 5 Department of Laboratory Medicine, National University Health System, Singapore; 6 Department of Infectious Diseases, Tan Tock Seng Hospital and National Centre for Infectious Diseases, Singapore; 7 Division of Infectious Diseases, Department of Medicine, National University Health System, Singapore

## Abstract

**Importance:**

Acute ischemic stroke (AIS) is a known neurological complication in patients with respiratory symptoms of COVID-19 infection. However, AIS has not been described as a late sequelae in patients without respiratory symptoms of COVID-19.

**Objective:**

To assess AIS experienced by adults 50 years or younger in the convalescent phase of asymptomatic COVID-19 infection.

**Design, Setting, and Participants:**

This case series prospectively identified consecutive male patients who received care for AIS from public health hospitals in Singapore between May 21, 2020, and October 14, 2020. All of these patients had laboratory-confirmed asymptomatic COVID-19 infection based on a positive SARS-CoV-2 serological (antibodies) test result. These patients were individuals from South Asian countries (India and Bangladesh) who were working in Singapore and living in dormitories. The total number of COVID-19 cases (54 485) in the worker dormitory population was the population at risk. Patients with ongoing respiratory symptoms or positive SARS-CoV-2 serological test results confirmed through reverse transcriptase–polymerase chain reaction nasopharyngeal swabs were excluded.

**Main Outcomes and Measures:**

Clinical course, imaging, and laboratory findings were retrieved from the electronic medical records of each participating hospital. The incidence rate of AIS in the case series was compared with that of a historical age-, sex-, and ethnicity-matched national cohort.

**Results:**

A total of 18 male patients, with a median (range) age of 41 (35-50) years and South Asian ethnicity, were included. The median (range) time from a positive serological test result to AIS was 54.5 (0-130) days. The median (range) National Institutes of Health Stroke Scale score was 5 (1-25). Ten patients (56%) presented with a large vessel occlusion, of whom 6 patients underwent intravenous thrombolysis and/or endovascular therapy. Only 3 patients (17%) had a possible cardiac source of embolus. The estimated annual incidence rate of AIS was 82.6 cases per 100 000 people in this study compared with 38.2 cases per 100 000 people in the historical age-, sex-, and ethnicity-matched cohort (rate ratio, 2.16; 95% CI, 1.36-3.48; *P* < .001).

**Conclusions and Relevance:**

This case series suggests that the risk for AIS is higher in adults 50 years or younger during the convalescent period of a COVID-19 infection without respiratory symptoms. Acute ischemic stroke could be part of the next wave of complications of COVID-19, and stroke units should be on alert and use serological testing, especially in younger patients or in the absence of traditional risk factors.

## Key Points

**Question:**

Is the risk of acute ischemic stroke (AIS) elevated in patients in the convalescent phase of an asymptomatic COVID-19 infection?

**Findings:**

In this case series of 18 male adults aged 50 years or younger who presented with AIS during the convalescent phase of an asymptomatic COVID-19 infection confirmed by a positive SARS-CoV-2 serological (antibodies) test result, the median onset of stroke was 2 months after the diagnosis of COVID-19.

**Meaning:**

Results of this study suggest a persistent increased risk of AIS in individuals with asymptomatic COVID-19 months after serological diagnosis, warranting stroke units to be on alert and use SARS-CoV-2 serological testing.

## Teaser

This case series reports the risk factors, incidence rate, and features of acute ischemic stroke experienced by a group of male patients aged 50 years or younger in the convalescent stage of COVID-19.

## Introduction

As the COVID-19 pandemic progresses, many asymptomatic or mildly symptomatic cases of COVID-19 infection have been identified, either by contact tracing^1,2,3^ or through surveillance programs, in various risk-stratified population groups.^4,5,6^ At the same time, there have been reports of symptoms emerging or persisting long after the resolution of the original acute infection, which have been described as long-haul symptoms of COVID-19.^7^

Acute ischemic stroke (AIS) is a known neurological complication in patients with acute COVID-19 infection.^8,9^ The mechanism of AIS that is associated with COVID-19 has been postulated to be secondary to an associated coagulopathy^10^ either by antiphospholipid antibodies^11^ or endotheliopathy.^12^ This theory has been observed in critically ill patients and in younger patients presenting with a large vessel occlusion.^13^ However, it is unknown whether patients who had an asymptomatic or a minimally symptomatic COVID-19 infection are similarly at risk for AIS as those patients who had overt acute respiratory COVID-19 illness.

In the city-state of Singapore, COVID-19 has been confirmed in 57 889 individuals as of October 14, 2020.^14^ Most of the infections have been localized to clusters of workers from South Asian countries (India and Bangladesh) who were living in dormitories,^14,15^ accounting for 94% (54 485 cases) of all COVID-19 cases in Singapore. This clustering was primarily associated with the proximity of the inhabitants in residential complexes. Active surveillance of close contacts has identified many cases of COVID-19 through serological tests. In this case series, we assessed AIS that occurred in a series of men aged 50 years or younger in the convalescent phase of asymptomatic COVID-19 infection.

## Methods

In this case series, all patients who experienced AIS and were under the care of public health care institutions in Singapore from May 21 to October 14, 2020 (a total of 21 weeks), were identified prospectively. These patients were admitted or referred to neurology units for the care of their AIS.^16^ This study was approved by the Singhealth Centralised Institutional Review Board, which granted a waiver of informed consent because of the observational nature of the study. We followed the Strengthening the Reporting of Observational Studies in Epidemiology (STROBE) reporting guideline.^17^

We retrieved clinical course, imaging, and laboratory data from the electronic medical records of each participating hospital. Acute ischemic stroke was confirmed by neuroimaging using either computed tomography angiography and computed tomography of the brain or magnetic resonance imaging and magnetic resonance angiography of the brain. Included patients had asymptomatic or no respiratory symptoms of COVID-19, which was confirmed by a positive SARS-CoV-2 serological (antibodies) test result. Serological testing was performed using either the Architect SARS-CoV-2 IgG assay (Abbott Diagnostics) or the Elecsys Anti-SARS-CoV-2 assay (Roche Diagnostics), which are immunoassays designed to detect the nucleocapsid antibody of SARS-CoV-2. All patients were managed by their respective neurologists, and the tests performed were according to the physician’s discretion. Patients were excluded if they had ongoing respiratory symptoms or positive SARS-CoV-2 test results confirmed through reverse transcriptase–polymerase chain reaction (RT-PCR) nasopharyngeal swabs.

We calculated the annual incidence rate of AIS for this study population and compared it with the annual incidence rate of a historical, national ischemic stroke cohort (obtained from the Singapore Ministry of Health) that was matched by age, sex, and ethnicity (Indian and Bangladeshi).^18^ The population at risk in the study cohort was the total number of confirmed COVID-19 cases (n = 54 485) within the dormitory population in Singapore.

### Statistical Analysis

Rate ratio (95% CI) and significance were calculated with the statistical test described by Rothman,^19^ in which a 2-sided, unpaired *P* < .05 was used to indicate statistical significance. Stata release 16 (StataCorp LLC) was used for the statistical analysis.

## Results

A total of 18 consecutive male patients, with a median (range) age of 41 (35-50) years (Table 1), presented with AIS as the initial but delayed manifestation of COVID-19. Seventeen patients were asymptomatic for acute respiratory illness but were diagnosed with COVID-19 (by a positive SARS-CoV-2 serological test result) before their AIS. One patient (6%) was tested during an acute hospital stay for AIS. One patient (6%) reported mild diarrhea during the time of isolation but had no respiratory symptoms or anosmia. All patients were tested (and had negative results) at least once for COVID-19 using RT-PCR swabs because they were either in direct contact with individuals with COVID-19 infection or had stayed in the same dormitory facilities as others with COVID-19 infection.

**Table 1.** Patient Demographic Characteristics, Medical History, and COVID-19–Related Information

Patient No.	Age range, y	Medical history	COVID-19 symptom	Chest radiograph result	No. of days from positive result to thrombotic event (serological test used)
1	36-40	None	None	Normal	40 (Elecsys Anti-SARS-CoV-2 assay; Roche)
2	36-40	HPT	None	Normal	19 (Elecsys Anti-SARS-CoV-2 assay; Roche)
3	41-45	None	Diarrhea	Normal	36 (Architect SARS-CoV-2 IgG assay; Abbott)
4	46-50	None	None	Normal	8 (Architect SARS-CoV-2 IgG assay; Abbott)
5	36-40	None	None	Normal	24 (Elecsys Anti-SARS-CoV-2 assay; Roche)
6	36-40	None	None	Normal	76 (Elecsys Anti-SARS-CoV-2 assay; Roche)
7	41-45	None	None	Normal	50 (Elecsys Anti-SARS-CoV-2 assay; Roche)
8	36-40	None	None	Normal	91 (Architect SARS-CoV-2 IgG assay; Abbott)
9	31-35	None	None	Normal	84 (Architect SARS-CoV-2 IgG assay; Abbott)
10	41-45	None	None	Normal	42 (Architect SARS-CoV-2 IgG assay; Abbott)
11	41-45	None	None	Normal	55 (Architect SARS-CoV-2 IgG assay; Abbott)
12	46-50	None	None	Normal	0 (Elecsys Anti-SARS-CoV-2 assay; Roche)^a^
13	46-50	HLD	None	Normal	96 (Elecsys Anti-SARS-CoV-2 assay; Roche)
14	46-50	HPT	None	Normal	130 (Architect SARS-CoV-2 IgG assay; Abbott)
15	31-35	None	None	Normal	108 Architect SARS-CoV-2 IgG assay; (Abbott)
16	41-45	Diabetes	None	Normal	54 (Architect SARS-CoV-2 IgG assay; Abbott)
17	36-40	HPT	None	Normal	64 (Architect SARS-CoV-2 IgG assay; Abbott)
18	41-45	HLD	None	Normal	113 (Architect SARS-CoV-2 IgG assay; Abbott)

Abbreviations: HLD, hyperlipidemia; HPT, hypertension.

^a^ Serological test performed during acute stroke hospitalization.

All patients survived with no evidence of respiratory symptoms during their AIS hospitalization. All patients had negative nasopharyngeal and pharyngeal RT-PCR swab results for COVID-19 during their acute hospitalization for AIS.

The median (range) time from positive serological result to AIS was 54.5 (0-130) days. Chest radiographs were unremarkable in all patients, and 12 patients (67%) had no known preexisting risk factors of AIS (ie, hypertension and hyperlipidemia). The spectrum and severity of stroke varied among the 18 cases, with a median (range) National Institutes of Health Stroke Scale score of 5 (1-25) (Table 2). Six patients (33%) had intravenous thrombolysis and/or endovascular therapy. Ten patients (56%) had a large vessel occlusion, including 7 patients (39%) who had an anterior circulation large vessel occlusion that was detected on presentation through neuroradiological imaging, and thrombectomy was performed in 5 of 10 patients (50%).

**Table 2.** Acute Ischemic Stroke Features and Cardiac Investigations^a^

Patient No.	Clinical symptoms of ischemic stroke	Stroke location	Stroke classification^b^	Treatment	NIHSS score on admission	NIHSS score 24 h after admission	Pretreatment brain imaging	Large vessel occlusion location	Transthoracic echocardiography findings
1	Right hemiplegia, dysarthria, and aphasia	Left insula and lentiform	Undetermined	Recombinant tPA and EVT	23	26	CTB and CTA	Left proximal MCA	Normal chamber sizes, EF 55%-60%, and no RWMA; no intracardiac shunt or mass
2	Right-sided ataxia, hemiplegia, and dysarthria	Left internal capsule	Small vessel occlusion	Recombinant tPA	5	1	MRI brain and MRA	None	Normal chamber sizes, EF 55%-60%, and no RWMA; no intracardiac shunt or mass
3	Dysarthria, vertigo, and unsteady gait	Bilateral cerebellar	Undetermined	Medical therapy	2	1	MRI brain and MRA	None	Normal chamber sizes, EF 55%-60%, and no RWMA; no intracardiac shunt or mass
4	Left hemiplegia	Right internal capsule	Small vessel occlusion	Medical therapy	9	9	MRI brain and MRA	None	Normal chamber sizes, EF 55%-60%, and no RWMA; no intracardiac shunt or mass
5	Left-sided numbness, hemiplegia, left hemianopia, and dysarthria	Right PCA territory	Undetermined	Medical therapy	10	12	MRI brain and MRA	Right PCA	Normal chamber sizes, EF 55%-60%, and no RWMA; no intracardiac shunt or mass
6	Right hemiplegia, dysarthria, global aphasia, and right-sided neglect	Left MCA territory	Undetermined	EVT	25	20	CTB and CTA	Left TICA	Normal chamber sizes, EF 55%-60%, and no RWMA; no intracardiac shunt or mass
7	Right hemiplegia and aphasia	Left MCA territory	Cardioembolism	Recombinant tPA and EVT	19	Intubated and sedated	CTB and CTA	Left TICA and distal left MCA	Left ventricular thrombus seen, EF 50%, and akinetic segment at the apex
8	Left hemiplegia, dysarthria, and left-sided neglect	Right MCA territory	Undetermined	EVT	12	8	CTB and CTA	Right proximal MCA	Normal chamber sizes, EF 55%-60%, and no RWMA; no intracardiac shunt or mass
9	Right hemiplegia and aphasia	Left MCA territory	Undetermined	Recombinant tPA and EVT	19	Intubated and sedated	CTB and CTA	Left TICA	Normal chamber sizes, EF 55%-60%, and no RWMA. No intracardiac shunt or mass
10	Left facial droop and left hemiplegia	Bilateral MCA territory	Undetermined	Medical therapy	2	0	CTB and CTA	Bilateral ICA but with intact intracranial	Normal chamber sizes, EF 55%-60%, and no RWMA; no intracardiac shunt or mass
11	Dizziness and unsteady gait	Left inferior cerebellum	Undetermined	Medical therapy	1	0	CTB and CTA	None	Normal chamber sizes, EF 55%-60%, and no RWMA. No intracardiac shunt or mass
12	Right hemiplegia and dysarthria	Left MCA territory	Undetermined	Medical therapy	6	6	CTB, MRI brain, and MRA	Left proximal MCA	Normal chamber sizes, EF 55%-60%, and no RWMA; no intracardiac shunt or mass
13	Left-sided ataxia and hemiplegia	Right MCA territory	Undetermined	Medical therapy	2	2	CTB, MRI brain, and MRA	None	Normal chamber sizes, EF 55%-60%, and no RWMA; no intracardiac shunt or mass
14	Right-sided weakness and ataxia	Left PCA territory	Undetermined	Medical therapy	5	5	CTB and CTA	Left PCA	Normal chamber sizes, EF 55%-60%, and no RWM; no intracardiac shunt or mass
15	Left-sided weakness and numbness	Right PCA territory	Cardioembolism	Medical therapy	1	1	MRI brain and MRA	Right PCA	Normal chamber sizes, EF 60%, and no RWMA; no intracardiac mass; patent foramen ovale seen and confirmed on TEE; TCD shows Spencer grade V right-to-left shunt
16	Dysarthria	Left frontal and right parietal	Cardioembolism	Medical therapy	1	0	CTB, MRI brain, and MRA	None	Normal chamber sizes, EF 65%, and no RWMA; no intracardiac shunt or mass; color turbulent flow seen across the interatrial septum because of presence of patent foramen ovale
17	Right monoparesis and dysphagia	Left caudate and lentiform nucleus	Undetermined	Medical therapy	5	1	CTB, MRI brain, and MRA	None	Normal chamber sizes, EF 65%, and no RWMA; no intracardiac shunt or mass
18	Ataxia	Left hemipontine	Undetermined	Medical therapy	1	0	CTB, MRI brain, and MRA	None	Normal chamber sizes, EF 55%-60%, and no RWMA; no intracardiac shunt or mass

Abbreviations: CTA, computed tomography angiography; CTB, computed tomography of the brain; EF, ejection fraction; EVT, endovascular therapy; ICA, internal cerebral artery; MCA, middle cerebral artery; MRA, magnetic resonance angiography; MRI, magnetic resonance imaging; NIHSS, National Institutes of Health Stroke Scale; PCA, posterior cerebral artery; RWMA, regional wall motion abnormalities; TCD, transcranial doppler; TEE, transesophageal echocardiography; TICA, terminal internal cerebral artery; tPA, tissue plasminogen activator.

^a^ All patients had normal sinus rhythm based on 48 to 72 hours of telemetry.

^b^ Stroke classification was based on the Trial of Org 10172 in Acute Stroke Treatment (TOAST) classification.

An extensive evaluation of the etiological factors in AIS was performed for all patients (Table 2). Three patients (17%) had a postulated cardiogenic cause of stroke: left ventricular thrombus and patent foramen ovale. Four patients (22%) had elevated homocysteine levels. In particular, 1 patient had high levels of homocysteine (93 μmol/L), suggesting a possible genetic origin of hyperhomocysteinemia (Table 3). All other patients had normal cardiac function without an alternative cardiac source of embolism and no atrial fibrillation detected on at least 48 hours of telemetry. Two patients (11%) were deemed to have had a small vessel occlusion as the subtype of the stroke, whereas 3 patients (17%) were considered to have had cardioembolism in accordance with the Trial of Org 10172 in Acute Stroke Treatment (TOAST) classification^20^ (Table 2). Eight patients (44%) were diagnosed with a large vessel occlusion but had no obvious atherosclerosis, and the cause of stroke was classified as undetermined.

**Table 3.** Hematological and Biochemistry Laboratory Test Results

Patient No.	White blood cell count, 4.0-10.0, × 10^9^/L^a^	Total neutrophils, 2.0-7.5, × 10^9^/L^a^	Total lymphocytes, 1.0-3.0, × 10^9^/L^a^	Total monocytes, 0.20-0.80, × 10^9^/L^a^	Platelet count, 140-450, × 10^9^/L^a^	Hemoglobin, 13.0-17.0, g/dL^a^	Albumin, 37-51, g/L^a^	LDH, 222-454, U/L^a^	Serum ferritin, 32-294, μg/L^a^	Homocysteine, 5.0-15.0, μmol/L^a^
1	16.0	13.5	1.9	0.53	227	14.5	38	324	127	4.0
2	5.5	3.3	1.4	0.60	257	14.9	42	340	135	17
3	6.1	3.8	1.6	0.52	262	15.2	40	326	196	34
4	5.9	3.8	1.4	0.51	299	14.2	39	194	Not performed	10
5	8.8	5.1	2.8	0.76	331	14.1	44	519	104	9.0
6	8.1	5.5	1.8	0.66	301	16.1	34	440	175	18
7	6.8	4.1	1.9	0.44	293	16.5	30	2387	Not performed	12
8	7.9	6.0	1.3	0.50	372	16.4	42	517	Not performed	93
9	9.2	5.5	2.9	0.50	322	13.2	43	446	Not performed	8.0
10	7.6	4.0	2.5	0.47	290	13.9	43	Not performed	Not performed	34.9
11	10.9	7.0	3.3	0.58	211	16.0	39	Not performed	Not performed	13.1
12	10.2	8.0	1.6	0.61	252	14.5	44	Not performed	Not performed	12.8
13	8.3	4.6	2.8	0.58	284	13.8	40	Not performed	Not performed	Not performed
14	9.1	5.3	2.6	0.74	211	18.6	39	Not performed	102	17.0
15	7.3	4.5	1.8	0.50	270	16.3	43	Not performed	Not performed	27.3
16	8.8	5.7	2.1	0.60	374	10.2	30	200	Not performed	12.6
17	8.3	5.5	2.4	0.44	229	17.5	41	Not performed	Not performed	10.3
18	8.0	5.1	2.3	0.54	299	15.9	39	Not performed	Not performed	18.5

Abbreviation: LDH, lactate dehydrogenase.

SI conversion factors: To convert hemoglobin grams per deciliter to grams per liter, multiply by 10.0; LDH units per level to microkatals per liter, multiply by 0.0167.

^a^ Reference ranges.

A screen for underlying coagulopathy was performed for all patients, and increased levels of dimerized plasmin fragment D (D-dimer) were detected in 3 patients (17%; Table 4). Two patients (11%) had positive results for lupus anticoagulant antibodies but negative results for other antiphospholipid antibodies (Table 5). Although not universally tested, von Willebrand factor antigen levels were elevated in 2 of 3 patients tested (Table 5).

**Table 4.** Coagulation Test Results^a^

Patient No.	INR, 0.9-1.1	PT, 9.5-11.5, s	aPTT, 24.0-34.0, s	Fibrinogen, 1.8-4.8, g/L	D-dimer, <0.5, mg/L FEU	ESR, 3-15, mm/h
1	1.0	13.3	25.1	2.7	3.2	1
2	0.9	12.7	25.6	3.1	<0.5	1
3	1.0	12.0	27.8	3.4	<0.5	5
4	1.0	10.6	31.5	Not performed	0.6	Not performed
5	0.9	11.9	26.5	6.2	1.0	10
6	1.0	13.3	23.3	3.0	1.0	1
7	1.0	10.8	21.6	3.3	5.2	8
8	1.0	11.9	26.9	2.5	4.9	Not performed
9	1.2	11.9	25.3	1.6	1.8	Not performed
10	1.1	12.9	29.6	Not performed	Not performed	26
11	1.1	11.1	29.7	3.5	0.6	Not performed
12	1.0	11.4	28.3	Not performed	<0.5	9
13	0.9	9.9	25.6	Not performed	<0.5	Not performed
14	1.2	11.8	30.1	3.4	0.5	17
15	1.0	11.4	38.8	3.9	<0.5	6
16	1.4	16.3	37.0	Not performed	Not performed	79
17	1.0	12.8	31.9	Not performed	Not performed	10
18	1.0	13.0	28.8	Not performed	Not performed	Not performed

Abbreviations: aPTT, activated partial thromboplastin time; D-dimer, dimerized plasmin fragment D; ESR, erythrocyte sedimentation rate; FEU, fibrinogen equivalent units; INR, international normalized ratio; PT, prothrombin time.

SI conversion factors: To convert D-dimer micrograms per milliliter to nanomoles per liter, multiply by 5.476.

^a^ The reference range or threshold value appears in the data-column heading of each test.

**Table 5.** Miscellaneous Laboratory Test Results^a^

Patient No.	Anticardiolipin IgG, <20, GPL unit/mL	Anticardiolipin IgM, <20, MPL unit/mL^a^	Lupus anticoagulant	Anti–β2-glycoprotein I IgM, SMU	von Willebrand factor antigen, 56-160, %^a^
1	<20	<20	Absent	Absent	260
2	<20	<20	Absent	Absent	134
3	<20	<20	Absent	Absent	Not performed
4	<20	<20	Absent	Absent	Not performed
5	<20	<20	Absent	Absent	Not performed
6	<20	<20	Absent	Absent	226
7	<20	<20	Absent	Absent	Not performed
8	<20	<20	Absent	Absent	Not performed
9	<20	<20	Absent	Absent	Not performed
10	<20	<20	Absent	Absent	Not performed
11	<20	<20	Absent	Absent	Not performed
12	<20	<20	Absent	Absent	Not performed
13	Not performed	Not performed	Not performed	Not performed	Not performed
14	<20	<20	Positive	Absent	Not performed
15	<20	<20	Absent	Absent	Not performed
16	<20	<20	Positive	Absent	Not performed
17	<20	<20	Absent	Absent	Not performed
18	<20	<20	Absent	Not performed	Not performed

Abbreviations: GPL, IgG phospholipid; MPL, IgM phospholipid; SMU, standard IgM anti–β2-glycoprotein I unit.

^a^ The reference range or threshold value appears in the data-column heading of each test that produces a numerical result.

The annual incidence rate of AIS in this all-male cohort with asymptomatic COVID-19 infection was 82.6 cases per 100 000 people. This rate was calculated on the basis of 18 patients who experienced AIS and a population at risk of 54 485 people over a study period of 21 weeks. The historical annual age-, sex-, and ethnicity-matched incidence rate of AIS was 38.2 cases per 100 000 people. This rate was calculated on the basis of 34 age-, sex-, and ethnicity-matched patients who experienced AIS from the 2018 national data and a similarly matched population at risk of 89 069 people over the same period.^21^ Therefore, the annual incidence rate in this all-male cohort was significantly higher compared with the annual incidence rate of the historical age-, sex-, and ethnicity-matched cohort (rate ratio, 2.16; 95% CI, 1.36-3.48; *P* < .001).

## Discussion

This case series highlights that adults 50 years or younger with asymptomatic or pauci-symptomatic COVID-19 infection diagnosed by positive SARS-CoV-2 serological test result may present with ischemic stroke during the convalescent phase of the infection. This finding underscores the value of SARS-CoV-2 serological testing during the etiological workup of patients who experienced AIS, given that a negative result from an RT-PCR test is expected during this period. Identifying antibodies to SARS-CoV-2 before AIS has public health implications, along with defining the complications associated with this emerging infection.

The patients in this series presented with AIS as the initial manifestation of COVID-19 infection a median of 54.5 days after the diagnosis was confirmed by a positive SARS-CoV-2 serological test result. This time from diagnosis to AIS was much longer compared with the time (estimated median of 16 days) for patients who presented with stroke and acute respiratory COVID-19 infection.^8,22^ In particular, 1 patient presented with a large vessel occlusion at 130 days after a positive SARS-CoV-2 serological test result, suggesting that the prothrombotic nature of COVID-19 may persist long after an acute infection is resolved.^10^ Moreover, the presence of these antibodies in younger adults coupled with the lack of traditional cardiovascular risk factors suggests an etiological association. The presence of SARS-CoV-2 antibodies in combination with other stroke risk factors also may hasten the manifestation of ischemic stroke.^23^ In addition, with an estimated production and persistence of COVID-19 antibodies about 2 weeks (although ranging from 1 to 6 weeks) after the initial COVID-19 infection,^24^ coagulopathy may likely be observed for months after the initial exposure in patients with a subclinical COVID-19 infection.^25^

In most of the cohort (56%), large vessel occlusions were detected on initial computed tomography or magnetic resonance angiography imaging, and 7 patients (39%) had an anterior circulation large vessel occlusion. This percentage was higher than an estimated 13% of patients in Hong Kong who had an ischemic stroke and an anterior circulation large vessel occlusion.^26^ Even in patients without a large vessel occlusion, the infarct pattern suggested an embolic phenomenon from a proximal source. Further evaluation did not reveal the origin of any proximal thrombus or any atherosclerotic-related lesions. These observations suggest an embolic or a prothrombotic phenomenon as the cause of AIS. Despite assessing a thorough stroke workup, we were unable to find an underlying mechanism except the unifying positive SARS-CoV-2 serological test results in all 13 patients (72%) whose stroke had an undetermined classification. The percentage (72%) of strokes classified as having an undetermined cause is higher than the estimated 20% to 25% cryptogenic stroke observed in Asian patients.^27^ In particular, a thorough cardiac workup showed that 1 patient with a left ventricular thrombus had no medical history or a medical reason to have a left ventricular thrombus. Again, the lack of an obvious source of thrombus formation suggested an etiological association with COVID-19.

The sustained prothrombotic mechanism in the convalescent phase of COVID-19 infection is currently uncertain and is an area of active research. In addition, the mechanism of stroke in patients with asymptomatic or mildly symptomatic COVID-19 infection likely differs from the mechanism in critically ill patients in the intensive care unit who have active respiratory COVID-19 infection. Overt inflammatory response and cytokine storm seen in critically ill patients with COVID-19 infection are factors in stroke through possible endothelial injury,^28^ in which elevated D-dimer levels have been found to be associated with arterial thrombotic events in patients with active COVID-19 infection.^29,30^ However, a recent coagulation study of patients 4 months after the resolution of respiratory COVID-19 infection demonstrated normal prothrombin time, fibrinogen level, D-dimer level, and von Willebrand factor antigen compared with levels in healthy control patients.^31^ This finding was consistent with the results of this case series, in which most patients had normal levels of these coagulation markers and only 3 patients had substantially elevated D-dimer levels. Sustained elevated levels of plasma factor VIII and plasminogen activator inhibitor 1 were found in the convalescent phase, partially explaining the hypercoagulable and hypofibrinolytic states.^31^ Tests for these coagulation markers were not routinely performed in clinical care and were not performed in this study cohort.

Overall, this case series revealed substantial heterogeneity in inflammatory and coagulation values across a spectrum of patients who experienced AIS in the convalescent period of asymptomatic COVID-19 infection. This finding suggests that the inciting prothrombotic mechanism of AIS in COVID-19 has yet to be defined in the convalescent phase and that current clinical inflammatory and coagulation markers are insufficient to identify individuals who are at risk for AIS and may only indicate the severity of the current stroke. Moreover, the variability of the tests performed by clinicians was also observed. Hence, to identify clinically useful and externally validated biomarkers for detecting arterial thrombosis, we propose testing a consistent panel of coagulation and inflammatory markers, which include plasma factor VIII or plasminogen activator inhibitor 1, across cohorts of patients with COVID-19 infection; these markers may be used prospectively and then subsequently repeated in the convalescent phase.

Antiphospholipid antibodies have been associated with AIS in patients with active respiratory COVID-19 infection.^11,29^ However, in the present case series, only 2 patients had lupus anticoagulant antibodies. Moreover, both of these patients had no other antiphospholipid antibodies, such as anticardiolipin IgM and IgG or anti–β2-glycoprotein I IgM. Although 1 patient had a large vessel occlusion seen on initial cerebral vascular imaging, 9 other patients with a large vessel occlusion did not have antiphospholipid antibodies. These results suggest that the presence of antiphospholipid antibodies is not commonly associated with the post–COVID-19 procoagulant state and may only be a partial risk factor in developing a large vessel occlusion in patients in the convalescent phase of COVID-19. Therefore, it is uncertain whether SARS-CoV-2 antibodies are factors in the persistent antiphospholipid response in the convalescent phase that is associated with AIS.

The observed AIS incidence rate (82.6 cases per 100 000 people) in this case series was 2.16 times higher than the national historical stroke incidence rate, resulting in an annual excess of 44 cases per 100 000 people. With the ongoing COVID-19 pandemic affecting more than 50 million persons worldwide at the time of this writing, the additional cases may translate to thousands of cases of strokes worldwide. This novel observation warrants confirmation in other locations with high volumes of unexplained AIS in younger adults with COVID-19 infection. Furthermore, future studies need to examine the association of COVID-19 with the increased risk of strokes in the older population, which may be potentially higher.

A previous study has shown that serological assays exhibit diagnostic accuracy for COVID-19 only after 14 days of symptom onset, allowing appropriate antibody seroconversion in the host.^32^ The present report suggests another suitable case-use criterion for COVID-19 serological tests, taking into consideration its natural history and clinical course of infection. Given that these tests are designed to be qualitative in nature, future research may identify the association between quantitative antibody titers and the severity of stroke.

The need for universal COVID-19 serological screening in younger adults without respiratory symptoms who experienced a stroke is debatable, and further studies are required to define the subgroups in whom and the duration in which a prothrombotic or persistent inflammatory state is particularly pronounced. The implications for patients who have recovered from a symptomatic COVID-19 infection and their risk of stroke during the convalescent phase are uncertain. Even if patients with asymptomatic COVID-19 infection were identified using serological tests, we may still be unable to ascertain the therapeutic implications for thrombotic events in these patients. The recent halting of recruitment for 3 trials of critically ill patients with COVID-19 because of lack of improved outcomes^33^ only demonstrates the lack of evidence for anticoagulation in COVID-19. In addition, the accuracy and limitations of existing serological methods for SARS-CoV-2 antibody detection have been openly debated and are an ongoing area of research and refinement.^34,35^ Nevertheless, with an uncertain incidence of asymptomatic COVID-19 infection in the population, only comprehensive population-based serological testing may reveal the extent of the seroconversion rate, enabling the estimation of the true association of COVID-19 with AIS.

Given the paucity of risk factors for stroke apart from the presence of SARS-CoV-2 antibodies, AIS could be part of the next wave of complications in the COVID-19 pandemic. Stroke units should be on high alert and administer serological testing, especially in younger adults or in the absence of traditional risk factors.

### Limitations

This study has limitations. First, it was purely observational in nature, and coagulopathy testing was dependent on physician discretion and the availability of clinical resources at each health care institution during the pandemic. This limitation was exemplified by the minimal testing for von Willebrand factor^36^ and other blood clotting factors, which are known coagulopathy risk factors in patients with COVID-19 infection. Second, all of the patients in this cohort were men from the South Asia region; most patients with COVID-19 infection in Singapore were living in the foreign worker dormitories with overwhelmingly male inhabitants. Hence, the findings related to AIS after COVID-19 infection may be generalizable only to a male South Asian population. In addition, the annual incidence rate was based on the single case series and should be interpreted with caution. A larger, population-based incidence rate is needed to verify the findings.

## Conclusions

This case series described 18 adults 50 years or younger who presented with AIS in the convalescent period of COVID-19 infection. The findings suggest an increased risk of AIS for these patients months after a serological diagnosis. Stroke may be the next wave of complications from COVID-19. Hence, stroke units should be on alert and use serological testing, especially in younger patients or in the absence of traditional risk factors.
